# Deep Learning Morphometric Analysis on Protocol Biopsies Predicts Future Graft Function

**DOI:** 10.1016/j.ekir.2026.106585

**Published:** 2026-05-06

**Authors:** Mira Ben Haberou, Patrick Bard, Jean-Baptiste Gibier, Hugo Gabriel Pereira De Almeida, Jamal Bamoulid, Mehdi Maanaoui, Alice Koenig, Maud Rabeyrin, Alexia Gazeu, Sami Ezzahid, Fanny Buron, Cécile Picard, Cécile Courivaud, Sophie Adrian Felix, Michel Paindavoine, Claire Tinel, Rémi Lenain, Laurent Martin, Georges Tarris, Manon Ansart, Mathieu Legendre

**Affiliations:** 1 Department of Nephrology, Centre Hospitalier Universitaire (CHU) Dijon, Dijon, France; 2 Skinet Team, Laboratoire d'Étude de l'Apprentissage et du Développement-French National Centre for Scientific Research (LEAD-CNRS), UMR 5022, Université de Bourgogne Europe, Dijon, France; 3 Department of Pathology, CHU Lille, Lille, France; 4 Department of Nephrology, CHU Besançon, Besançon, France; 5 Department of Pathology, CHU Besançon, Besançon, France; 6 Department of Nephrology, CHU Lille, Lille, France; 7 Department of Transplantation, Nephrology and Clinical Immunology, Hospice Civils de Lyon, Lyon, France; 8 Department of Pathology, Hospices civils de Lyon, Lyon, France; 9 Department of Pathology, CHU Dijon, Dijon, France

**Keywords:** artificial intelligence, deep learning, kidney biopsy, morphometry, prognosis, transplantation

## Abstract

**Introduction:**

The predictive value of Banff classification in protocol transplant biopsies without specific lesions is limited. Morphometry provides precise data on microstructures, surpassing semiquantitative scores but is time-consuming. This study evaluates whether automated morphometric analysis with deep learning can predict glomerular filtration rate at 3 years using machine learning.

**Methods:**

This retrospective study included kidney transplant recipients who underwent protocol biopsy without specific lesion. The models were trained and tested on the training/test cohort, with external validation on the application cohort. Eight deep learning algorithms extracted 23 morphometric parameters from whole-slide images (WSI). Ten machine learning models were tested for 3 years glomerular filtration rates prediction.

**Results:**

A total of 367 patients were included. The means of the 3-year estimated glomerular filtration rates (eGFR) were 53 ± 23 and 53 ± 22 ml/min per 1.73 m^2^ in the training/test and application cohorts, respectively. In the training/test cohort, eGFR correlated negatively with interstitial fibrosis (*r* = −0.33; *P* < 0.001), tubular atrophy (*r* = -0.39; *P* < 0.001), and artery luminal stenosis (*r* = −0.29; *P* < 0.001), and positively with glomerular density (*r* = 0.16; *P* < 0.05) and glomerular epithelial (*r* = 0.33; *P* < 0.001), endothelial (*r* = 0.30; *P* < 0.001), and mesangial (*r* = 0.25; *P* = 0.002) cell densities. Kernel Ridge and Bayesian models achieved the best predictions (mean absolute error [MAE] = 11 ± 1 ml/min per 1.73 m^2^). External validation showed good association between predicted with Bayesian model and observed eGFR (MAE = 13 ± 11 ml/min per 1.73 m^2^, *r* = 0.68; *P* < 0.001). After correction with Bland-Altman bias, paired analysis showed no significant difference between predicted and observed eGFRs (*P* = 0.953).

**Conclusion:**

Integrating automated morphometric analyses into machine learning models may help predict glomerular filtration rates 3 years after protocol biopsies.

## Graphical abstract

**Figure:**
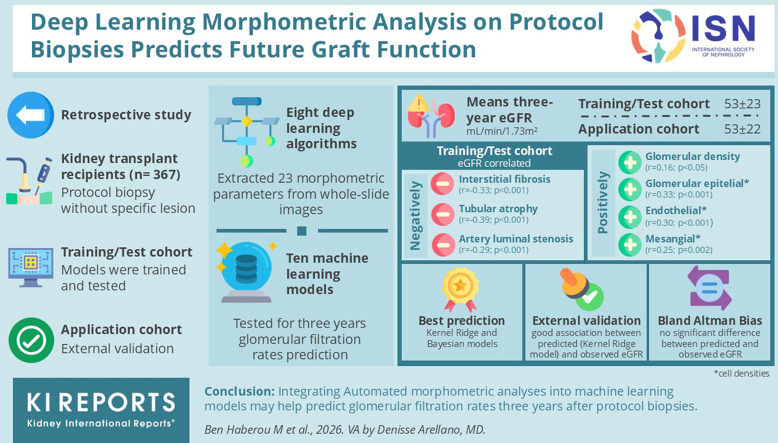

The months following the transplantation procedure are key for maintaining long-term graft survival. Early detection of immune, toxic, or infectious complications is necessary to adapt therapeutic strategies.^1^ To this end, some teams perform protocol biopsies to identify early signs of complications.^2^, ^3^, ^4^ These evaluations focus on semiquantitative gradings of structural damage, and are summarized in the international Banff classification.^5^^,^^6^ However, the Banff classification provides limited descriptions of microstructural morphology, which may strongly influence transplant survival. Moreover, when no specific lesions are identified, the biopsy’s predictive value for kidney prognosis remains unclear.

Morphometry consists of annotating WSIs to extract quantitative data.^7^ Morphometry allows obtaining continuous data, which is more precise than a semiquantitative grading. This technique evaluates the surface area, volumetric densities, or the shape of objects, elements that are not typically assessed in routine pathology.^7^^,^^8^ Denic *et al.*^9^, ^10^, ^11^ have shown associations between morphometric analyzes, such as glomerular volume, mesangial expansion, the percentage of interstitial fibrosis/tubular atrophy, with the risk of late graft failure. Even though computational pathology is becoming more common in transplant pathology, manual segmentation is too time-consuming for daily practice.

Artificial intelligence, particularly deep learning, could help for automating morphometric analysis.^12^, ^13^, ^14^ Instance segmentation, an advanced deep learning technology, delineates each object in a WSI at a pixel precision, assigning unique masks to individual objects.^12^ These analyses allow precise, rapid, and reproducible identifications of kidney structures. Our team has previously developed tools with instance segmentation, which can automate the evaluation of various morphometric analyses, such as the percentage of interstitial fibrosis/tubular atrophy and vascular intimal thickening, glomerular volume, and interstitial leukocyte density.^12^^,^^15^^,^^16^ We also found a correlation between glomerular architecture, such as endothelial cells’ relative area, and the risk of graft function decline.^17^

Data regarding kidney transplant morphometry are limited.^7^^,^^8^ Yi *et al.*^18^ have previously proven the impact of deep learning-based analysis of tubulo-interstitial area and leukocytes infiltration on the risk of graft loss. To our knowledge, no study specified in transplant pathology has evaluated a complete automated combined morphometric analysis of intraglomerular structures, interstitial, and vascular areas, nor have they evaluated their associated impact on kidney function. In this study, we examined the relationship between an extensive automated morphometry analysis and the evolution of eGFR. The objective was to determine whether an automated morphometric evaluation of protocol biopsies without specific lesions could predict eGFR at 3 years using machine learning.

## Methods

### Study Population

This retrospective study included patients who had undergone transplant biopsy between 3- and 6-months post-transplantation at the University Hospital of Besançon from 2012 to 2020, or at the University Hospital of Lille from 2015 to 2018, and at the University Hospital of Hospices Civils de Lyon from 2016 to 2018. Only adult recipients with ABO-compatible kidney transplants without acute or chronic rejection, probable antibody-mediated rejection, microvascular inflammation (2022 Banff Classification),^5^ viral nephropathy, *de novo* nephropathy, or recurrence of initial nephropathy on the protocol biopsy analyses were consecutively included. Patients were not excluded if the biopsy showed interstitial fibrosis/tubular atrophy, vascular intimal thickening, and/or arteriolar hyalinosis. A minimum follow-up period of 3 years after biopsy was mandatory.

Patients were excluded if they died within the first 3 years of follow-up. Other exclusion criteria included use of belatacept during follow-up, missing eGFR at the time of the biopsy and/or at 3 years (+/−3 months), poor-quality WSI, or insufficient biopsy material (fewer than 7 analyzable glomeruli and/or fewer than 1 medium-caliber vessel).

Only data from recipients were collected. Clinical and biological data were extracted from institutional medical records. These included age at transplantation, gender, initial nephropathy, history of diabetes or hypertension, immunosuppressive regimen, serum creatinine levels, and eGFR (chronic kidney disease – epidemiology formula) at the time of biopsy and at 3-, 5-, and 7-years post-transplantation. For patients on dialysis, eGFR was arbitrarily defined at 10 ml/min per 1.73 m^2^. Additionally, donor-specific antibodies before transplantation, BK polyomavirus replication in the blood at biopsy, and events during follow-up such as rejection episodes, BK virus infections, death, and dialysis were recorded.

The end of follow-up was defined as death, dialysis, or last eGFR available. The last follow-up visit allowed in this study was November 2023 at Besançon and June 2024 at Lille.

This study was conducted in accordance with the institutional ethics committee and the Helsinki Declaration. The clinical and research activities being reported are consistent with the Principles of the Declaration of Istanbul as outlined in the Declaration of Istanbul on Organ Trafficking and Transplant Tourism. All patients had given their oral consent before the study.

### Objectives

The main objective was to predict the eGFR at 3 years after biopsy. This prediction was based on morphometric data from protocol biopsies, combined with data from transplant recipients. This study used 2 distinct artificial intelligence models— deep learning for image segmentation and machine learning for predicting eGFRs.

**Figure 1:**
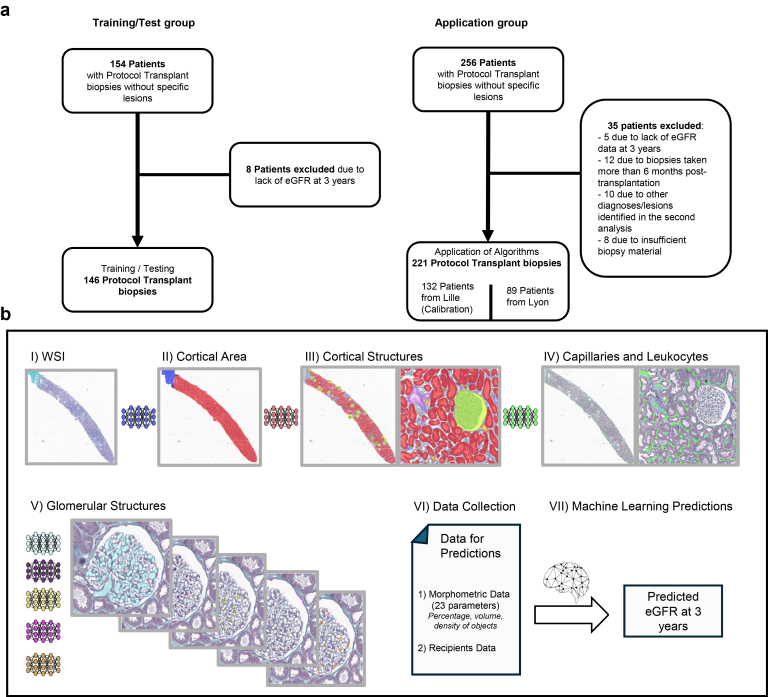
Study Protocol. (a) Flow charts for training/test and application cohorts. (b) Instance segmentation with a pipeline of eight successive deep learning algorithms to predict 3 years eGFR with machine learning algorithms. I) Masson’s trichrome protocol biopsies (×25 magnification) were digitized into whole-slide images (WSI) before deep learning analyzes. II) The first algorithm identified the capsular (Blue), cortical (Red), and medullary areas. III) Within the cortical area, a second algorithm segmented normal tubules (red), atrophic tubules (orange), Bowman’s capsule (yellow), nonsclerotic glomeruli tuft (light green), and globally sclerotic glomeruli (light blue), internal elastic lamina (pink), external elastic lamina (purple), vein (deep blue). IV) A third algorithm differentiated peritubular capillaries (green) and leukocytes (red). The final 5 Instance Segmentation algorithms focused on nonsclerotic glomeruli (×400 magnification). They segmented their capillaries (light blue). They also identified individual podocytes (orange), glomerular epithelial (pink), endothelial (purple), and mesangial cells (yellow). VI-VII) Clinical, biological, and morphometric analyzes were used to train, test (Training/Test cohort) and to evaluate in an external cohort (Application cohort) the ability to predict eGFR at 3 years of ten machine learning models. eGFR, estimated glomerular filtration rates; WSI, whole-slide images.

Patients were separated into 2 distinct groups. The first cohort (training/test cohort), consisting of patients from Besançon, was used to develop and test the machine learning prediction algorithms. The second cohort (application cohort), consisting of patients from Lille and Lyon, was used to evaluate the generalizability of these algorithms in predicting eGFR in external groups. The application cohort was then split depending on whether patients were from Lille or Lyon. To limit a center effect on histological morphometry in Besançon, patients from Lille were used for calibration of the predictions. A bias in eGFR predictions at 3 years with the main model was calculated using a Bland-Altman analysis. The value of the bias observed in the Lille cohort was then added to the eGFR predictions of patients from Lyon to evaluate whether it enhanced the predictions. The flowcharts are summarized in Figure 1. Secondary analyses focused on predicting eGFR at 5 and 7 years. As neural networks do not have proper formulas to determine sample sizes, we chose to include all the patients matching the inclusion criteria during the period of inclusion

### Histology

Kidney biopsies were formalin-fixed, paraffin-embedded, then cut into 2 μm sections, and stained with blue or green Masson’s trichrome. The digitization of sections was performed with a Nano-zoomer 2.0 HT (Hamamatsu, Japan, model C9600-12) or Aperio GT 450 D (Leica AG, Germany). A single section level was used per biopsy. Two kidney pathologists, with a French diploma in kidney pathology, independently performed a lesion overview and Banff classification for each biopsy, and then compared their results. In total, 3 kidney pathologists were involved in the review process. When discrepancies were present, the highest score was retained to ensure that no lesions potentially biasing the analysis were overlooked. If a biopsy reached a rejection or a microvascular inflammation score according to only 1 of the 2 pathologists, or when combining the most severe scores from each, the biopsy was excluded. Immunofluorescence data (including C4d) were extracted from the pathology report. No electron microscopy was performed routinely.

### Morphometric Data and Deep Learning

WSI were analyzed at 25x magnification with resolutions of 227 nm/pixel (Besancon) and 263 nm/pixel (Lille and Lyon). A pipeline of 8 successive deep learning algorithms was used for instance segmentation. We previously trained and tested these algorithms on transplants and native kidneys using the same methodology.^12^^,^^15^^,^^17^ None of the biopsies from the actual study were used for the training/testing of the algorithms.

For segmentation, we used the Mask R-CNN Inception ResNet V2 model (faster_rcnn_inception_resnet_v2_keras), implemented in Python with TensorFlow and Keras. The Mask R-CNN model was pretrained using the "TF2 Detection Zoo" repository (https://github.com/tensorflow/models/blob/master/research/object_detection/g3doc/tf2_detection_zoo.md).^12^^,^^15^^,^^17^ A Dell Precision 7920 Tower (073A) computer equipped with two Intel Xeon Gold 5120 14-Core processors (188 GB RAM) and an NVIDIA RTX A6000 graphics card (48 GB VRAM) were used in this study.

The sequence of the 8 algorithms was programmed to run automatically (Figure 1). The first algorithm identified the capsular, cortical, and medullary areas. Within the cortical area, a second algorithm segmented normal, atrophic tubules, nonsclerotic glomeruli, glomerular tuft, globally sclerotic glomeruli, veins, and arteries (differentiating internal, and external elastic laminas). Of note, nonsclerotic glomeruli were categorized differently depending on whether the vascular pole of their tuft was visible (complete glomeruli) or not (partial glomeruli).

Within the remaining unclassified “interstitial areas,” a third algorithm differentiated peritubular capillaries and leukocytes, distinguishing those located within the capillaries and those in the surrounding interstitial area. The final 5 algorithms focused on nonsclerotic glomeruli. They segmented their capillaries, podocytes, glomerular epithelial, endothelial, and mesangial cells.

We performed individual reviews of the inferences. When errors appeared in the predictions, the algorithms inferred WSIs *de novo*. To obtain parameters of interest, for each segmentation, both the number of objects and their surface areas were quantified. Supplementary Methods and Supplementary Table S1 summarize the 23 included morphometric parameters and their formulas.

### eGFR Prediction

Ten machine learning regression models were used on the training/test cohort including the following: Bayesian regression, elastic net, gradient boosting, kernel ridge, least absolute shrinkage and selection operator, orthogonal matching pursuit polynomial, random forest, ridge, support vector regression, and a classic multiple linear regression model.^19^ Additional descriptions of the predictive models are provided in the Supplementary Methods.

Cross-validation with the k-fold technique was used on the training/test cohort to develop and evaluate algorithms for eGFR predictions.^20^ This cross-validation randomly divides the dataset into “k” equal-sized subgroups. One group is used for testing (k), whereas the remaining groups are allocated for training. This process iterates multiple times (or folds), ensuring every sample is included in both training and testing. A 10-fold cross-validation model was chosen, with 10% of the patients designated for testing and 90% for training in each fold.^20^ Results obtained were the means of these 10 folds. For each machine learning model, the MAE was collected (Supplementary Methods).^21^

The application cohort intended to furnish external validation. We selected the model with the lowest MAE and standard deviation as the main model for this cohort. It used the previous algorithms to predict eGFR values. These predictions were then compared with the observed eGFR values.

Clinical and biological data used in the models were recipient age, male or female gender, diabetes, hypertension, DSA, BK virus replication, and eGFR at biopsy. The 23 morphometrics data used in the models are described in Supplementary Methods.

To refine our analysis, we also included the following 3 other versions of the predictive model: with histological data and eGFR at biopsy, with only histological data, and with clinical and biological data (including eGFR at biopsy). None of the included patients had missing data included in the models. Blinding was preserved, with no access to clinical or test results across assessors.

### Statistical Analyses

Quantitative and categorical data were expressed as mean ± standard deviation and *n* (%), respectively. The paired Wilcoxon test was used to compare predicted and observed eGFR values. Student’s *t*-test or Mann–Whitney U test compared 2 quantitative unpaired variables depending on whether the distribution was normal or not. Categorical variables were compared using the chi-square test (or Fisher’s exact test when appropriate). The correlation between 2 variables was evaluated using Pearson or Spearman correlation test, based on whether the distribution was normal or not. Bland-Altman analyses were used to assess biases of predictions. Receiver operating characteristics curves were constructed for the prediction of eGFR < 45 ml/min per 1.73 m^2^. Statistical analyses were performed with a bilateral alpha risk of 5% using GraphPad PRISM 6.01 software (GraphPad Software, La Jolla, CA) and IBM SPSS 23 (IBM, Armonk, NY). Machine learning analyses were performed in Python using the scikit-learn module (https://scikit-learn.org/stable/) version 1.5.0.

## Results

### Population

A total of 367 patients were included, with 146 patients from Besançon training/test cohort and 221 from application cohort (132 from Lille and 89 from Lyon University Hospitals) (Figure 1). Patients’ characteristics are described in Table 1. The mean age at biopsy was 53 ± 13 years, 231 (63%) patients were men. The mean eGFR at biopsy was 53 ± 19 ml/min per 1.73 m^2^. Patients from the training/test cohort were significantly older and more likely to have hypertension (both *P* < 0.001) (Table 1).

**Table 1:** Study population

Variables	All patients (*N* = 367)	Training/test cohort (*n* = 146)	Application cohort (*n* = 221)	*P*-value^a^
Clinical data				
Age (yrs)	53 ± 13	55 ± 13	52 ± 14	0.036^b^
Male gender, *n* (%)	231 (63)	89 (61)	142 (64)	0.522
History of hypertension, *n* (%)	293 (80)	134 (92)	159 (72)	< 0.001^b^
History of diabetes, *n* (%)	70 (19)	32 (22)	38 (17)	0.260
Initial nephropathy				
Diabetes and/or hypertension *n* (%)	66 (18)	25 (17)	41 (19)	0.727
Polycystic kidney disease *n* (%)	60 (16)	33 (23)	27 (12)	0.009^b^
Glomerulonephritis *n* (%)	50 (14)	28 (19)	22 (10)	0.012^b^
Tubulointerstitial nephritis *n* (%)	23 (6)	4 (3)	19 (9)	0.023^b^
Others *n* (%)	168 (46)	56 (38)	112 (51)	0.020^b^
Immunosuppressors				
Antithymocyte globulin induction, *n* (%)	155 (42)	31 (21)	124 (56)	< 0.001^b^
Basiliximab induction, *n* (%)	212 (58)	115 (79)	97 (44)	< 0.001^b^
Tacrolimus, *n* (%)	360 (98)	145 (99)	215 (97)	0.251
Mycophenolate mofetil, *n* (%)	352 (96)	145 (99)	207 (94)	0.008^b^
Corticosteroid, *n* (%)	299 (81)	146 (100)	153 (69)	< 0.001^b^
Biological data				
DSA at biopsy, *n* (%)	33 (9)	7 (4)	26 (11)	0.022^b^
BK virus Replication at Biopsy, *n* (%)	38 (10)	29 (20)	10 (5)	< 0.001^b^
Serum Creatinine at Biopsy (mg/dl)	1.5 ± 0.5	1.5 ± 0.5	1.5 ± 0.5	0.968
eGFR at Biopsy (ml/min per 1.73 m^2^)	53 ± 19	52 ± 20	53 ± 18	0.649
3 yrs eGFR (ml/min per 1.73 m^2^)	53 ± 22	53 ± 23	53 ± 22	0.860
5 yrs eGFR (ml/min per 1.73 m^2^)	51 ± 23 (*n* = 330)	50 ± 24 (*n* = 128)	52 ± 22 (*n* = 202)	0.431
7 yrs eGFR (ml/min per 1.73 m^2^)	50 ± 26 (*n* = 272)	51 ± 27 (*n* = 83)	49 ± 25 (*n* = 189)	0.486
Follow-up				
Follow-up (mos)	91 ± 29	85 ± 30	95 ± 23	< 0.001^b^
Rejection during follow-up, *n* (%)	38 (10)	8 (6)	30 (14)	0.014^b^
Dialysis at follow-up, *n* (%)	42 (11)	15 (10)	27 (12)	0.572
Death, *n* (%)	38 (10)	13 (9)	25 (11)	0.741

DSA, donor-specific antibodies; eGFR, estimated glomerular filtration rate.

Variables are expressed as mean ± SD.

a Student’s *t*-test or Mann–Whitney’s U-tests compared 2 quantitative unpaired variables depending on whether the distribution was normal or not. Categorical variables were compared using the chi-square test (or Fisher’s exact test when appropriate).

b Significant *P*-values.

The training/test cohort and application cohort included respectively 146 (100%) and 214 (97%) deceased donor transplants, with 102 (70%) and 188 (85%) first transplants, and 7 (5%) patients in Besançon with DSA at biopsy, 26 (12%) patients in Lyon and Lille with DSA at biopsy. The cohorts were similar in clinical profiles, but immunosuppressive regimens were different between groups. Basiliximab was given to 115 (79%) of the training/test patients versus 97 (44%) of the application patients. For maintenance, all training/test patients were on corticosteroids at biopsy, compared with 153 (69%) in the application group.

Mean eGFRs at 3 years were respectively of 53 ± 23 and 53 ± 22 ml/min per 1.73 m^2^ for the training/test and application cohorts. During the first 3 years of follow-up, 5 (3%) patients (2 rejections, 2 BK polyomavirus infections, 1 progressive decline) from the training/test cohort and 9 (4%) patients (7 progressive declines, 1 from T-cell mediated rejection, and 1 hemolytic and uremic syndrome) from the application cohort started chronic dialysis. During this period, 4 (3%) patients (3 acute T-cell-mediated and 1 acute antibody-mediated rejections) from training/test cohort and 28 (8%) patients (16 borderline, 6 acute T-cell-mediated, 4 acute antibody-mediated, 1 chronic active antibody-mediated, and 1 chronic T-cell-mediated rejections) from application cohort experienced a rejection. Two (1%) patients from training/test and 11 (3%) from application cohorts suffered from BK polyomavirus biopsy proven nephropathy. Follow-up time was longer in patients in the application cohort, therefore, the number of rejections during follow-up was higher (Table 1).

### Histology and Morphometry

Table 2 presents the Banff Classification evaluation of protocol biopsies for each group. Most biopsies showed no arterial (v0 in all patients), tubular (t0 in *n* = 361, 98 %), interstitial, capillary (i0 and ptc 0 in *n* = 363 and 364, 99%), or glomerular (g0 in *n* = 333, 91%) proliferation.

**Table 2:** Visual and automated histological data

Variables	All patients (*N* = 367)	Training/test cohort (*n* = 146)	Application cohort (*n* = 221)	*P*-value^f^
Banff score^a^				
Glomerulitis g score ≥ 1, *n* (%)	33 (9)	3 (2)	31 (14)	< 0.001^g^
Transplant glomerulopathy cg score ≥ 1, *n* (%)	3 (1)	1 (1)	2 (1)	0.819
Interstitial inflammation *i* score ≥ 1, *n* (%)	5 (1)	2 (1)	3 (2)	0.986
Tubulitis *t* score ≥ 1, *n* (%)	6 (2)	2 (1)	4 (2)	0.325
Peritubular capillaritis ptc score ≥ 1, *n* (%)	3 (1)	1 (1)	2 (1)	0.819
Arteriolar hyalinosis ah score ≥ 1, *n* (%)	211 (57)	72 (49)	139 (63)	0.013^g^
Intimal thickening cv score ≥ 1, *n* (%)	173 (47)	44 (30)	129 (58)	< 0.001^g^
Intimal arteritis v score ≥ 1, *n* (%)	0 (0)	0 (0)	0 (0)	0.999
IF/TA score, *n* (%)				
1	169 (46)	63 (43)	106 (48)	0.393
2	12 (3)	5 (3)	7 (3)	0.999
3	2 (1)	1 (1)	1 (1)	0.999
Morphometric parameters^b^				
Non sclerotic glomeruli (%)	92 ± 12	89 ± 14	94 ± 9	< 0.001^g^
Mean glomerular volume (μm^3^)	6.0 ± 2.7 × 10^6^	5.4 ± 2.5 × 10^6^	6.3 ± 2.7 × 10^6^	< 0.001^g^
Glomerular density (/mm^3^^c^)	12 ± 6	11 ± 6	12 ± 6	0.010^g^
Artery luminal stenosis (%)	31 ± 22	30 ± 22	31 ± 21	0.677
Mean tubular area (μm^2^)	3065 ± 842	2965 ± 760	3131 ± 887	0.111
Tubular atrophy (%)	23 ± 10	26 ± 9	21 ± 9	< 0.001^g^
Interstitial fibrosis (%)	22 ± 6	22 ± 6	22 ± 6	0.610
Relative peritubular capillaries area (% of cortical Area)	7 ± 2	7 ± 1	8 ± 1	0.002^g^
Mean number of leukocytes per peritubular capillary	0.2 ± 0.1	0.2 ± 0.1	0.1 ± 0.1	< 0.001^g^
Number of leukocytes in the most affected capillary	4 ± 2	5 ± 2	3 ± 1	< 0.001^g^
Capillary occlusion by leukocytes (%)	2 ± 1	3 ± 1	2 ± 1	< 0.001^g^
Interstitial leukocytes density (cells/mm^3^^d^)	271711 ± 92225	343683 ± 86122	224165 ± 59606	
Mean mesangial Area (μm^2^)	7911 ± 2636	7173 ± 2913	8399 ± 2316	< 0.001^g^
Mean parietal cells area (μm^2^)	39 ± 6	33 ± 3	42 ± 5	< 0.001^g^
Mean parietal cells density (cells/mm^3eδ^)	68235 ± 31831	94131 ± 26642	51127 ± 21881	< 0.001^g^
Mean podocytes area (μm^2^)	39 ± 6	35 ± 4	43 ± 5	< 0.001^g^
Mean podocytes density (cells/mm^3^^e^)	97842 ± 38402	125959 ± 36082	79267 ± 26909	< 0.001^g^
Mean endothelial cells area (μm^2^)	21 ± 3	18 ± 1	22 ± 2	< 0.001^g^
Mean endothelial cells density (cells/mm^3^^e^)	193190 ± 75042	217445 ± 87507	177167 ± 60605	< 0.001^g^
Mean mesangial cells area (μm^2^)	22 ± 3	19 ± 1	24 ± 3	< 0.001^g^
Mean mesangial cells density (cells/mm^3^^e^)	147089 ± 67734	206380 ± 54272	107919 ± 42628	< 0.001^g^
Mean glomerular capillary area (μm^2^)	115 ± 23	102 ± 15	123 ± 24	< 0.001^g^
Relative capillary area (% of glomerulus)	27 ± 5	29 ± 4	26 ± 5	< 0.001^g^

IF/TA, interstitial fibrosis/tubular atrophy.

Variables are expressed as Mean± Standard Deviation.

a Assessed by 2 pathologists according to the 2022 classification 5.

b Parameters included in models, assessed by neural networks.

c Relative to cortical volume.

d Relative to interstitial volume.

e Relative to glomerular volume.

f Student’s *t*-test or Mann–Whitney’s U-test compared 2 quantitative unpaired variables depending on whether the distribution was normal or not. Categorical variables were compared using the chi-sqaure test (or Fisher’s exact test when appropriate).

g Significant *P*-values.

The mean inference time per biopsy was about 1hour 30 minutes for automated segmentation. Table 2 presents morphometric data. Means percentages of interstitial fibrosis/tubular atrophy and intimal fibrosis were 22 ± 8%, and 31 ± 22%, respectively. The mean glomerular volume was 2.2 ± 3.1 × 10^6^ μm^3^, glomerular volumetric density was 12 ± 6 glomerulus/mm^3^, interstitial leukocytes volumetric density was 271711 ± 92225 cells/mm^3^ of interstitial volume, relative peritubular capillaries area 7 ± 2% of cortical area, and glomerular endothelial volumetric density was 193190 ± 75042 cells/mm^3^ of glomerular volume. Of note, patients of the training/test cohort had more tubular atrophy and less nonsclerotic glomeruli. Glomerular cells (except for endothelial density) and capillary densities were significantly higher in the training/test cohort compared with the application cohort (Table 2).

### Training/Test Cohort

In univariate analyses, several morphometric parameters were associated with eGFR at 3 years in the training/test cohort. As expected, eGFR was negatively correlated with percentages of interstitial fibrosis (*r* = -0.33; *P* < 0.001), tubular atrophy (*r* = −0.39; *P* < 0.001), and artery luminal stenosis (*r* = -0.29; *P* = 0.001). Additionally, eGFR was associated with percentage of peritubular capillary occlusion (*r* = −0.19; *P* = 0.022), mean tubular surface area (*r* = 0.17, *P* < 0.05), glomerular density (*r* = 0.16; *P* < 0.05), and glomerular epithelial (*r* = 0.33; *P* < 0.001), endothelial (*r* = 0.30; *P* = 0.001), and mesangial (*r* = 0.25; *P* = 0.002) cells densities, and the relative area of glomerular capillaries (*r* = 0.22; *P* = 0.008). Morphometric evaluations according to eGFR stages at 3 years are described in Supplementary Figure S1.

Eleven predictive models were trained and tested. Table 3 presents the performances of the predictive models for eGFR at 3 years. Among the models, Bayesian and Kernel Ridge predictions showed the highest accuracy in the main version combining morphometric and clinical-biological data. These 2 models achieved MAEs of 11 ± 1 ml/min per 1.73 m^2^ with both R^2^ of 0.56. Interestingly, this performance was comparable to that of the version using morphometric data combined with eGFR at biopsy (Table 3). The prediction versions using only morphometric or only clinical-biological data had MAEs of respectively 16 ± 1 and 15 ± 2 ml/min per 1.73 m^2^ in the best models (Bayesian models). In the main version combining all data, predictions with Kernel Ridge models for eGFR at 5 (*n* = 128) and 7 (*n* = 83) years achieved MAEs of 14 ± 1 and 15 ± 3 ml/min per 1.73 m^2^, respectively (Supplementary Tables S2 and S3).

**Table 3:** Prediction performance of the 3-year eGFR by machine learning models tested on 146 patients from the training/test cohort

Models	Morphometric, clinical and biological data	Morphometric data and eGFR at biopsy	Morphometric data	Clinical and biological data
	MAE ± SD	MAE ± SD	MAE ± SD	MAE ± SD
Bayesian	11.5 ± 1.1	11.5 ± 1.0	16.0 ± 1.1	14.9 ± 1.6
Elastic net	11.8 ± 1.1	11.6 ± 1.1	16.5 ± 1.8	15.1 ± 1.6
Gradient boosting	12.6 ± 0.9	12.8 ± 1.1	16.8 ± 0.8	15.5 ± 1.4
Kernel Ridge	11.4 ± 1.2	11.5 ± 0.8	16.4 ± 1.5	17.5 ± 1.9
LASSO	11.8 ± 1.1	11.7 ± 1.0	16.5 ± 1.8	15.2 ± 1.6
OMP	11.6 ± 1.0	11.7 ± 1.2	16.6 ± 1.6	15.3 ± 1.6
Polynomial	14.1 ± 1.5	12.1 ± 1.0	16.4 ± 2.3	15.2 ± 1.6
Random forest	12.1 ± 1.0	12.3 ±0.9	16.4 ± 1.0	15.3 ± 1.6
Ridge	11.9 ± 1.1	11.8 ± 1.0	16.4 ± 2.0	14.8 ± 2.0
SVR	13.0 ± 1.2	12.7 ± 1.3	15.7 ± 1.3	15.8 ± 1.4
Multiple linear	12.6 ± 1.3	12.1 ± 1.0	16.4 ± 2.2	15.2 ± 1.6

LASSO, least absolute shrinkage and selection operator; MAE, mean absolute error in ml/min per 1.73m^2^; OMP, orthogonal matching pursuit polynomial; SVR^,^ support vector regression.

### Application Cohort

**Figure 2:**
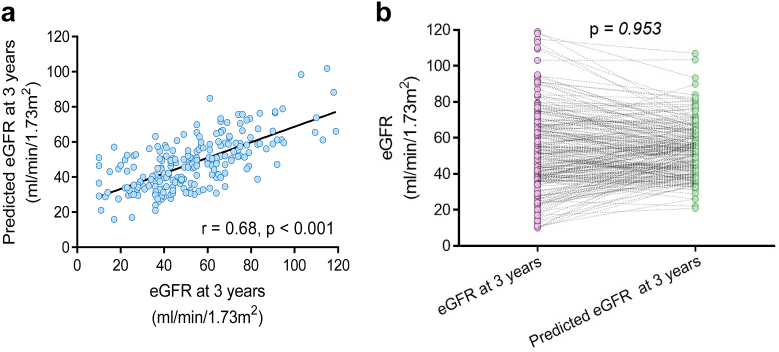
Evaluation of a Kernel Ridge model in the application cohort to predict eGFR at 3 years. a: The correlation between the observed eGFR at 3 years and the one predicted was assessed by Pearson correlation test. b: Comparison between predicted and observed eGFR values using the paired Wilcoxon test. Predicted eGFR was calibrated using the mean bias value (5 ml/min per 1.73 m^2^) from Bland-Altman analysis. Each small, dashed line represents connections between the same patient. eGFR, estimated glomerular filtration rates.

**Figure 3:**
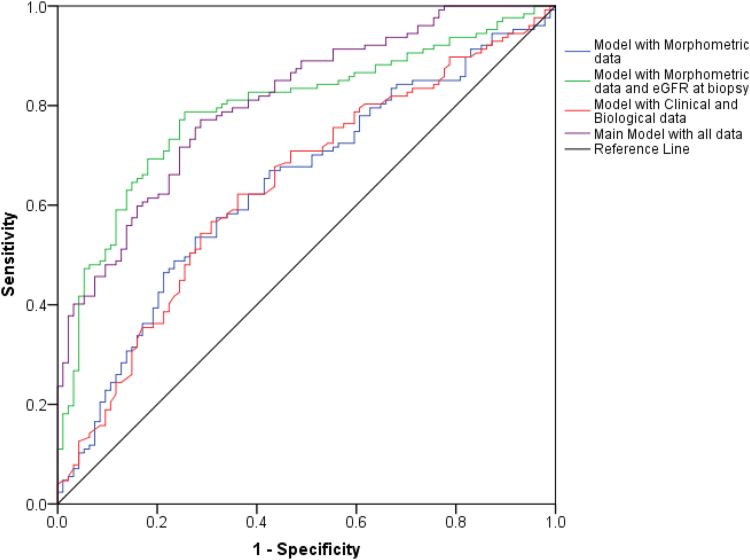
Receiver Operating Characteristic curves assessing the capacity of algorithms to predict an eGFR < 45 ml/min per 1.73 m^2^ at 3 years. The area under the Receiver operating characteristic curves were: 0.81 (95% confidence interval [CI]: [0.75–0.86], *P* < 0.001) for the Bayesian model with morphometric, clinical and biological data (purple), 0.79 (95% CI: [0.73–0.85], *P* < 0.001) for the Random Forest model with morphometric data and eGFR at Biopsy (green), 0.64 (95% CI: [0.57–0.71], *P* < 0.001) for the Random Forest model with morphometric data (blue), and 0.64 (95% CI: [0.57–0.71], *P* < 0.001) for the linear regression model with clinical and biological data (red). eGFR, estimated glomerular filtration rates.

All previously trained prediction models were applied to the application cohort (Table 4). The lowest MAE was observed with the Bayesian Regression model (13 ± 11 ml/min per 1.73 m^2^). The mean 3 years observed and predicted (Bayesian regression model) eGFRs were 53 ± 22 ml/min per 1.73 m^2^ and 47 ± 15 ml/min per 1.73 m^2^, respectively. A significant correlation was observed between predicted and observed eGFRs (*r* = 0.68; *P* < 0.001) (Figure 2a). The area under the receiver operating characteristic curve for the prediction of 3-year eGFR < 45 ml/min per 1.73 m^2^ with the Bayesian model was 0.81 (95% Confidence Interval [0.75–0.86], *P* < 0.001). Figure 3 presents receiver operating characteristic curves of different models for the predictions at 3 years. Of note, the MAE was not modified when evaluated only in patients with deceased donors (*n* = 214), nor in patients with a rejection and/or BK polyomavirus nephropathy within the first 3 years (*n* = 34), or those without (*n* = 187), but it was lower in living donors (*n* = 7; 17 ml/min per 1.73 m^2^). The MAE was lower with the Kernel Ridge model (15 ± 12 ml/min per 1.73 m^2^). Prediction performances of the different models are described in Table 4. As expected, with paired analyses, predicted eGFR at 3 years with the Bayesian model was significantly different from initial eGFR at biopsy (*P* < 0.001).

**Table 4:** Prediction performance of the 3-year eGFR by machine learning models tested on 221 patients from the application cohort

Models	Morphometric, clinical and biological data			Morphometric data and eGFR at biopsy			Morphometric data			Clinical and biological data		
	MAE ± SD	r	*P*-values	MAE ± SD	r	*P*-values	MAE ± SD	r	P-values	MAE ± SD	r	*P*-values
Bayesian	13.3 ± 10.8	0.68	< 0.001	13.5 ± 10.9	0.67	< 0.001	20.0 ± 16.8	0.12	0.052	16.7 ± 12.6	0.35	< 0.001
Elastic net	13.4 ± 10.8	0.68	< 0.001	13.3 ± 10.9	0.68	< 0.001	20.3 ± 16.8	0.21	0.002	16.7 ± 12.7	0.35	< 0.001
Gradient Boosting	13.5 ± 11.2	0.62	< 0.001	13.5 ± 11.3	0.61	< 0.001	17.4 ± 13.1	0.24	< 0.001	16.8 ± 12.8	0.32	< 0.001
Kernel Ridge	15.5 ± 11.6	0.66	< 0.001	15.0 ± 11.5	0.66	< 0.001	25.2 ± 19.2	0.19	0.006	17.6 ± 14.2	0.27	< 0.001
LASSO	13.8 ± 10.9	0.68	< 0.001	13.1 ± 10.8	0.68	< 0.001	22.8 ± 17.8	0.21	0.002	16.7 ± 12.6	0.35	< 0.001
OMP	14.0 ± 11.0	0.67	< 0.001	13.4 ± 10.9	0.68	< 0.001	20.7 ± 16.5	0.22	< 0.001	16.7 ± 12.7	0.35	< 0.001
Polynomial	30.8 ± 23.1	0.24	< 0.001	14.6 ± 11.9	0.64	< 0.001	24.1 ± 18.9	0.29	< 0.001	16.5 ± 12.9	0.36	< 0.001
Random Forest	13.4 ± 10.2	0.66	< 0.001	13.5 ± 10.4	0.65	< 0.001	16.5 ± 13.4	0.33	< 0.001	17.2 ± 13.5	0.33	< 0.001
Ridge	15.1 ± 11.3	0.67	< 0.001	15.4 ± 11.7	0.66	< 0.001	23.9 ± 18.5	0.21	0.002	16.6 ± 12.7	0.36	< 0.001
SVR	26.2 ± 36.2	0.39	< 0.001	13.2 ± 11.0	0.66	< 0.001	17.3 ± 14.2	0.28	< 0.001	16.8 ± 12.9	0.30	< 0.001
Multiple Linear	13.7 ± 10.9	0.66	< 0.001	14.6 ± 11.8	0.64	< 0.001	24.1 ± 19.7	0.29	< 0.001	16.5 ± 12.9	0.36	< 0.001

LASSO, least absolute shrinkage and selection operator; MAE, mean absolute error in ml/min per 1.73 m^2^; OMP, orthogonal matching pursuit polynomial; SVR, support vector regression.

Predictions were less performant at 5 (*n* = 202) and 7 (*n* = 189) years (best MAE of 15 ± 12 with Bayesian Regression and 16 ± 13 ml/min per 1.73 m^2^ with Random Forest, respectively) (Supplementary Tables S4 and S5).

At 3 years, the best MAEs were 13 ± 10 ml/min per 1.73 m^2^ for the model with histological data and eGFR at biopsy (Random Forest), and 16 ± 13 ml/min per 1.73 m^2^ for the model with histological data alone (Random Forest) and 16 ± 13 with only clinical-biological data (Linear Regression) (Table 4).

### Calibration

**Figure 4:**
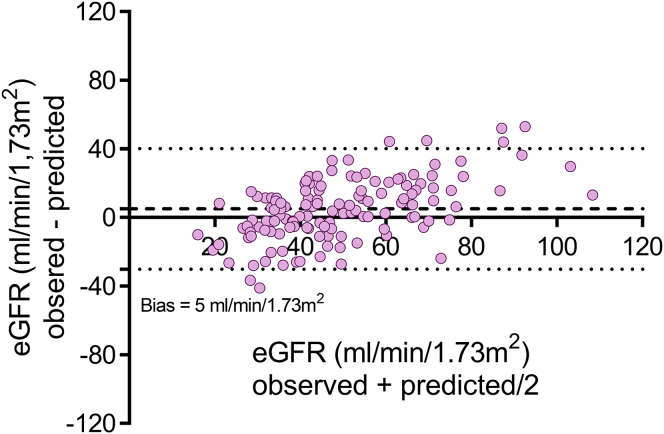
Bland-Altman plot between lesions observed and predicted in the calibration cohort from Lille (*n* = 132). Bland-Altman plot showing a bias of 5 ml/min per 1.73 m^2^ between eGFRs observed and predicted. The mean bias is represented by the big dashed black lines with the 95% limits of agreement represented by the small, dashed lines. eGFR, estimated glomerular filtration rates.

The subgroup of patients from Lille (*n* = 132) was used to calibrate predictions with a Bland-Altman analysis. Before calibration, the predicted eGFR with the Bayesian model was significantly lower than the one observed at 3 years (paired analyses, *P* < 0.001). In this group, Bland-Altman analysis showed a mean bias of 5 ml/min per 1.73 m^2^ (95% Confidence Interval: [−30 to 40]) (Figure 4). After adjusting predictions with the bias, paired analysis showed no significant difference between predicted and observed values (*P* = 0.879). Of note, the MAE at 3 years in this subgroup was 14 ± 11 ml/min per 1.73 m^2^.

Bias values were then added to the predictions at 3 years for patients from Lyon (*n* = 89). After adjusting predictions, paired analysis showed no significant difference between predicted and observed values (*P* = 0.729). The MAE went from 11 ± 9 to 10 ± 9 ml/min per 1.73 m^2^. Comparison of patients from Lille and Lyon is described in Supplementary Table S6.

In all patients from the application cohort (*n* = 221), after adjusting predictions, paired analysis showed no significant difference between predicted and observed values (*P* = 0.953) (Figure 2b). The MAE remained at 13 ± 10 ml/min per 1.73 m^2^. After adding the bias value, no statistical differences were found between observed and predicted eGFR values with the Bayesian model at 5 years.

## Discussion

Our study shows the potential of automated morphometric analysis for transplant protocol biopsies. We combined a freely available deep learning-based segmentation of WSI with clinical and biological data, which were subsequently analyzed using machine learning algorithms. As a result, our tool was able to predict eGFR at 3 years with a MAE ranging from 11 to 13 ml/min per 1.73 m^2^.

Even though an elevation in serum creatinine level is often the most common predictive tool to evaluate the risk of kidney function deterioration, only relying on it would lead to delayed management.^22^ Thus, earlier tests are necessary to target patients at risk of eGFR deterioration. The iBox and the “Kidney Transplant Failure Score,” 2 predictive models, combine histology, donor-recipient, and immunological data to predict transplant prognosis.^23^, ^24^, ^25^ Although their results are promising, they have limitations because of the sole use of the Banff classification in histological evaluation. The Banff classification only focuses on inflammatory or fibrotic lesions, lacks precision, and has medium to poor interobserver reproducibility.^26^, ^27^, ^28^ Our study focuses on detailed and automatic quantification of multiple morphometric parameters. This method using deep learning enhances reproducibility, provides precise analyses, and is suitable for daily practice.^13^^,^^14^

In the training/test cohort, the Kernel Ridge and Bayesian prediction models achieved the best performances. However, the MAE of 11 ± 1 ml/min per 1.73 m^2^ remained imperfect. This limitation shows the complexity of predicting eGFR evolution. First, as we decided to focus our analysis on the impact of morphometric analysis, some clinical and biological data such as proteinuria were not recorded.^29^ Our sample size was too small to include many nonhistological variables. Second, our study relied solely on characteristics from the recipient but many factors contributing to transplant dysfunction are linked to donor characteristics.^23^^,^^30^^,^^31^ Moreover, although few patients experienced rejection episodes or BK virus nephropathy during follow-up, the possible effect of undiagnosed rejections, recurrences of nephropathies, and comorbidity-related episodes cannot be excluded.

When the Bayesian model was applied to the external application cohort, its performance was slightly reduced. In the training/test cohort, both trained and tested patients were from the same center. This could partly explain the better predictions in this cohort. Additionally, the evaluations techniques were not strictly equivalent between the 2 cohorts, as k-fold cross-validation was used for the training/test cohort. Numerous significant differences appeared between groups. Indeed, patients from the training cohort were significantly older, had different immunosuppressive therapies, a higher number of tubular atrophy lesions, a lower rate of permeable glomeruli, and higher volumetric densities for all tested cell types, with smaller cell areas. These differences are probably linked to variations in transplant populations across regions, as well as differing transplantation policies and immunosuppressive treatment strategies between centers. Variations may also exist in the preparation of biopsies at the 2 pathology centers.^32^ Such factors could explain the relative shrinking or expansion of certain glomerular structures observed. These differences between groups, particularly in morphometric evaluations, may have impacted predictions and limited the generalizability of our algorithm. It highlights the influence of population heterogeneity on morphometry-based models. Without calibration, the MAE remained at levels close to that of the training set, but the predicted eGFR was significantly different. After calibration, this difference in independent cohorts disappeared completely at 3, 5, and 7 years of follow-up. This improvement suggests that such approaches may help adjust predictions across centers.

As the number of patients experiencing end-stage kidney disease during follow-up was too low to use it as an end point, we used surrogate markers such as eGFR at 3, 5, and 7 years. It should be noted that eGFR variation has been shown to be strongly correlated with the risk of end-stage kidney disease.^33^, ^34^, ^35^, ^36^ We felt that 3-year eGFR may be sufficiently distant from transplantation to not be heavily influenced by transient factors such as ischemia–reperfusion injury, delayed graft function, infectious diseases, urological problems, and early immunosuppressive adjustments. However, previous subclinical lesions, such as progressive tubulointerstitial remodeling, microvascular rarefaction, or early glomerular structural alterations may impact kidney function. It also remains close enough to transplantation that targeted interventions could still impact the course of graft dysfunction. Most protocol biopsies are classified as having no specific lesions. We therefore considered it valuable to use these apparently unnecessary biopsies to predict kidney function over time. Even without hard end points, predicting midterm GFR is clinically relevant. It helps identify patients at risk of significant GFR decline, guides stage-dependent preventive strategies in chronic kidney disease, and may support monitoring and adjustment of immunosuppressive therapies.

Biopsies with rejection or viral nephropathy lesions were not included. These restrictive criteria limited the number of inclusions but allowed for a more precise evaluation of the impact of the donor kidney’s architecture. The predictive performance of the main model with clinical characteristics was very close to the version using only morphometric data and eGFR at biopsy. These results suggest that morphometric parameters had a real impact on predictions. However, the eGFRs at biopsy were close to those at 3 years. One could hypothesize that the only real factor leading to the predictions was eGFR at biopsy. Nonetheless, the predictions using only morphometric data were very close to the predictions using clinical-biological data with eGFR at biopsy. Moreover, predicted eGFR was significantly different than eGFR at biopsy.

Our team and others had previously demonstrated the potential effect of morphometry on transplant outcomes. We observed that low glomerular endothelial cells’ area, as well as low epithelial cell volumetric density, were associated with higher risks of graft function deterioration.^17^ Denic *et al.*^9^^,^^37^ observed a correlation between manual glomerular volumes, percentages of interstitial fibrosis, tubular atrophy, and artery luminal stenosis with transplant outcomes. In this study, except for glomerular volume, we confirmed these associations, as well as glomerular and cell densities, with the risk of decreased eGFR.^9^^,^^17^^,^^37^ Our univariate analysis also identified additional potential markers for kidney function deterioration such as peritubular capillary occlusion. Our study integrates multivariable analyses of all these markers and thus evaluates their interaction effects on kidney function.

One of the strengths of this study is the workflow involving multiple deep learning algorithms with 23 morphometric parameters analyzed per biopsy. To our knowledge, only Hölscher *et al.*^8^ had previously developed a similar large-scale histomorphometry deep learning framework. However, their study included only a few transplant biopsies without specific lesions. Their study mainly focused on the morphometry of IgA nephropathy in native kidney biopsies.^8^ Even though our studies are centered on different topics, they both confirm that deep learning can automate morphometric analyses. Indeed, manual segmentation is too time-consuming for daily practice.^7^

In this study, most of the models for eGFR predictions used machine learning algorithms.^38^ These algorithms are able to integrate multiple complex and nonlinear data.^39^^,^^40^ Unlike most classical statistical analyses, they use some notions of uncertainty and offer a more flexible approach to searching for associations between factors.^41^^,^^42^ Kernel Ridge and Bayesian models showed the best 3-year predictions. Thanks to its kernel functions, which are well-suited for analyzing complex nonlinear relationships, the Kernel Ridge model can be used to highlight subtle and nonobvious interactions.^43^ The Bayesian model allows for the integration of previous information and provides a probabilistic estimation of predictions.^44^ By predicting eGFR, we tried to go beyond simple outcomes like graft survival or loss. This approach delivers an in-depth view of kidney function over time. However, the performance of the models may have been limited by the large number of included parameters. We chose to test several machine learning predictive models in addition to classical multilinear models. Most of the time, all prediction capacities were close to that of the classical linear model. The aim was not to rule out linear models, but rather to find the most precise algorithm to predict eGFR, with the lowest range of errors.

This study has some limitations. First, the predictive performance of our algorithms remained moderate, which limits their use in clinical practice. Our results show that automated morphometric analysis captures a significant and independent signal associated with long-term kidney function. Rather than replacing current clinical prediction tools, this approach is intended to complement them by providing objective and reproducible freely available histological quantification. Future studies integrating morphometric features with established clinical predictors are likely to further improve predictive accuracy and better define the clinical utility of such tools. Moreover, we did not compare our results with previous predictive published models because of differences in end points between our studies, and the lack of donor data.^23^, ^24^, ^25^^,^^29^ We also did not compare the morphometric analysis with a model including the Banff classification, as most patients had no lesion. Other studies have previously shown that morphometric analysis of interstitial fibrosis and mesangial expansion, were more strongly associated with transplant function decline than the Banff classification.^10^^,^^11^^,^^45^ We also did not compare manual segmentation to deep learning predictions. Indeed, our algorithms had already been externally validated.^15^^,^^17^ However, some small prediction errors persisted, such as misidentification at tissue borders. Several missing data, such as infectious episodes, limit the interpretation of our results. Stratification of patients without intercurrent events would have allowed a more refined analysis. Only a very low number of patients had a kidney transplant from a living donor, and none were from the training cohort, limiting the applicability of our algorithms in these patients. Patients and kidney biopsies were not equivalent between groups.

Another limitation is that patients who died within 3 years were excluded, which may have removed the most severe patients from the analysis. However, as we expected to predict kidney function at 3 years, a 3-year follow-up was mandatory. Patients treated with belatacept were also excluded. Indeed, belatacept is known to significantly influence kidney function after withdrawal of calcineurin inhibitors.^46^ It is possible that some patients with severe fibrotic lesions were excluded because they would have received belatacept. Nonetheless, it should be noted that the algorithm’s predictions are likely not applicable to patients once they are treated with belatacept. Although these exclusions helped improve the accuracy of predictions, they may limit the generalizability of our findings and should be considered when applying the algorithm.

Deep learning with instance segmentation can automate morphometric analyses. Its predictions are associated with eGFR 3 years after the protocol biopsy. Integrating automated morphometric analyses into machine learning models could help predict eGFR progression.

## Disclosure

All the authors declared no competing interests.

## Data Availbility

The 8 deep learning weight files are available at: https://github.com/SkinetTeam. The predictions models with weight files are available at: https://github.com/skinet-team/.

## Supplementary Material

Supplementary file (PDF) **Supplementary Methods. Supplementary References. Figure S1.** Heatmap of histomorphometric parameters from the Training/Test cohort (*n* = 146) according to the chronic kidney disease stage at 3 years. **Table S1.** Formulas for parameters of interest. **Table S2.** Prediction performance of the 5-year eGFR by Machine Learning models tested on 128 patients from the Training/Test cohort. **Table S3.** Prediction performance of the 7-year eGFR by Machine Learning models tested on 83 patients from the Training/Test cohort. **Table S4.** Prediction performance of the 5-year eGFR by Machine Learning models tested on 202 patients from the Application cohort. **Table S5.** Prediction performance of the 7-year eGFR by Machine Learning models tested on 189 patients from the Application cohort. **Table S6.** Application cohort. STARD Checklist.

## Footnotes

Supplementary file (PDF)

**Supplementary Methods.**

**Supplementary References.**
**Figure S1.** Heatmap of histomorphometric parameters from the Training/Test cohort (*n* = 146) according to the chronic kidney disease stage at 3 years. **Table S1.** Formulas for parameters of interest. **Table S2.** Prediction performance of the 5-year eGFR by Machine Learning models tested on 128 patients from the Training/Test cohort. **Table S3.** Prediction performance of the 7-year eGFR by Machine Learning models tested on 83 patients from the Training/Test cohort. **Table S4.** Prediction performance of the 5-year eGFR by Machine Learning models tested on 202 patients from the Application cohort. **Table S5.** Prediction performance of the 7-year eGFR by Machine Learning models tested on 189 patients from the Application cohort. **Table S6.** Application cohort. STARD Checklist.
