# Supplementary material for: Deep Learning Morphometric Analysis on Protocol Biopsies Predicts Future Graft Function
Source: Kidney Int Rep. 2026 May 6;11(7):106585. doi: 10.1016/j.ekir.2026.106585 (PMC13265662; doi:10.1016/j.ekir.2026.106585)
Supplement: Supplementary file (PDF) — Supplementary Methods. Supplementary References. Figure S1. Heatmap of histomorphometric parameters from the Training/Test cohort (n = 146) according to the chronic kidney disease stage at 3 years. Table S1. Formulas for parameters of interest. Table S2. Prediction performance of the 5-year eGFR by Machine Learning models tested on 128 patients from the Training/Test cohort. Table S3. Prediction performance of the 7-year eGFR by Machine Learning models tested on 83 patients from the Training/Test cohort. Table S4. Prediction performance of the 5-year eGFR by Machine Learning models tested on 202 patients from the Application cohort. Table S5. Prediction performance of the 7-year eGFR by Machine Learning models tested on 189 patients from the Application cohort. Table S6. Application cohort. STARD Checklist. [file mmc1.pdf]

SUPPLEMENTAL MATERIALS

TABLE OF CONTENT

SUPPLEMENTAL METHODS **2**

Parameters and Models 2

SUPPLEMENTAL TABLES **5**

Supplemental Table S1: Formulas for parameters of interest 5

Supplemental Table S2: Prediction performance of the 5-year eGFR by Machine Learning models tested on 128 patients from the Training/Test cohort 6

Supplemental Table S3: Prediction performance of the 7-year eGFR by Machine Learning models tested on 83 patients from the Training/Test cohort 6

Supplemental Table S4: Prediction performance of the 5-year eGFR by Machine Learning models tested on 202 patients from the Application cohort 7

Supplemental Table S5: Prediction performance of the 7-year eGFR by Machine Learning models tested on 189 patients from the Application cohort 7

Supplemental Table S6: Application cohort **8**

Supplemental Figure S1: Heatmap of histomorphometric parameters from the Training/Test cohort (n=146) according to the chronic kidney disease stage at three years **9**

SUPPLEMENTAL REFERENCES **9**

SUPPLEMENTAL METHODS

Parameters and Models

To train, test and evaluate our models, we used automated morphometric parameters, as well as biological and clinical data at biopsy. The morphometric parameters were obtained from Deep Learning analysis of protocol transplant biopsies. Formulas of these parameters are described in Supplemental Table 1.

The 23 morphometric parameters used in the models were:

- Non sclerotic glomeruli (% of total glomeruli)
- Mean glomerular volume (μm^3)
- Glomerular volumetric density (/mm^3 of cortical volume)
- Artery luminal stenosis (%)
- Mean tubular area (μm^2)
- Tubular atrophy (%)
- Interstitial fibrosis (%)
- Relative peritubular capillaries area (% of cortical area)
- Mean number of leukocytes per peritubular capillary
- Number of leukocytes in the most affected capillary
- Capillary occlusion by leukocytes (%)
- Interstitial volumetric leukocytes density (cells/mm^3 of interstitial volume)
- Mean mesangial area (μm^2)
- Means podocytes, parietal, endothelial, and mesangial cells areas (μm^2)
- Means parietal endothelial, and mesangial cells volumetric densities (cells/mm^3 of glomerulus volume)
- Mean glomerular capillary area (μm^2)
- Relative capillary area (% of glomerulus)

The biological parameters used in the models were:

- Presence of Donor Specific Antibodies at biopsy
- Presence of BK virus replication at biopsy
- eGFR value (ml/min/1.73m^2) at biopsy

The clinical parameters at biopsy used in the models were:

- Age
- Sex
- History of diabetes
- History of hypertension

Four models were trained, tested and evaluated at 3,5 and 7 years after protocol biopsy:

1. A model with automated morphometric parameters, as well as biological and clinical data at biopsy
2. A model with histological data and eGFR at biopsy
3. A model with only histological data
4. A model with only clinical data and biological data

Predictive models

For the development of eGFR predictive model, 10 different models were first chosen depending on whether their theoretical principles were more in line with our objective. Each of them was then trained and evaluated according to a predefined metric to make a subsequent selection based on the results. The chosen models are Bayesian Regression, Elastic Net,

Gradient Boosting, Kernel Ridge, Least Absolute Shrinkage and Selection Operator, Orthogonal Matching Pursuit Polynomial, Random Forest, Ridge, Support Vector Regression, and classic multiple linear regression.
Bayesian Regression is a kind of linear regression in which regression coefficients are not supposed to be fixed like in classical (frequentist) linear regression but are allowed to vary according to prior probability distribution. The goal is to model an ensemble of behavior of the input variable. In addition, confidence intervals can be outputted.
Elastic Net is another generalization of linear regression including regularization terms. Specifically, it makes use of L1- (mean of absolute value) and L2-norm (quadratic mean). It prevents the model from diverging in case of a great number of highly uncorrelated features.
Gradient Boosting falls under ensemble learning techniques. The idea is to improve weak classifiers by iteratively adding new ones for correcting weakness of the preceding. Weak classifiers are classically Regression Trees. Regression Tree is an algorithm that uses binary decision for each input feature to construct a tree whose leaves are the predicted value.
If Ridge Regression is a weaker regularized generalization of linear regression which only uses L2-norm; Kernel Ridge generalizes Ridge Regression to non-linear cases. It makes use of a function named kernel which maps non-linear input into a linear space in which linear regression can be applied.
Least Absolute Shrinkage and Selection Operator (LASSO) is a generalization of linear regression which uses only L1-norm. Where Ridge regression tends to globally lower the absolute values, it rather tends to set unnecessary parameters to zero and thus makes the model less complex and less prone to overfitting.
The objective of Orthogonal Matching Pursuit Polynomial is to represent input data as a combination of orthogonal polynomial that can thus be used to output regression value.
Random Forest is another instance of ensemble learning techniques where Regression Trees are aggregated. The goal is to overcome Regression Trees' drawback to overfitting. Unlike Gradient Boosting, aggregation is done by averaging or voting scheme depending on the application.
Like classic multiple linear regression, the core of Support Vector Regression is to determine a hyperplane that best fits the data. Yet, unlike the former, it does not compute errors for each data point but ignores some of them, located in a small margin around the hyperplane, in order to focus on the more important ones. The aim is to improve robustness to outliers and noise. Support Vector Regression can also be used in combination with kernel function to handle non-linear data.

Models' evaluation relies on Mean Absolute Error (MAE), whose definition is given by the following equation:

$$\mathrm{MAE} = \frac{1}{\mathrm{n}}\sum_{1=0}^{\mathrm{n}} |\mathrm{y_i} - \mathrm{\hat{y}_i}|$$

where n is the number of input data, $\mathrm{y_i}$ and $\mathrm{\hat{y}_i}$ are respectively the ground truth and the predicted value for the ith input.

Training and evaluation are carried out using a process known as K-fold cross-validation. A hyperparameter, designated by K, is defined, in this case set to 10. K times, the training/test cohort is divided into a training set and a test set, provided that the test set never coincides. Training and evaluation of each model are conducted K times, and final evaluation result consists of the average of the K MAE for each model. This is a common method, in the case of small datasets, to overcome the dependence of predictions on random data selection
Development of the predictive models was done on a computer with the following features:
- Model: Dell Precision 3581
- Processor: 13th Gen Intel(R) Core (TM) i9-13900H (2.60 GHz)
- Memory: 32,0 Go
- Graphical card: NVIDIA RTX 2000 Ada Generation Laptop GPU
- Operating system: Windows 11

- Python: 3.12.4
- Scikit-learn: 1.5.0
- SciPy: 1.14.0

SUPPLEMENTAL TABLES

Supplemental Table S1: Formulas for parameters of interest

Parameters	Formulas	Description
**Percentage of Non-Globally Sclerotic Glomeruli**	$100x\ \frac{Total\ number\ of\ Non\ sclerotic\ Glomeruli}{Total\ number\ of\ Glomeruli}$	
**Mean glomerular area (*Aglom*)** (S1)	$\frac{Aglom\_complete + Aglom\_partial}{total\ number\ of\ non-sclerotic\ glomeruli}$	Aglom_complete= areas of Tufts of complete glomeruli<br>Aglom_partial= areas of Tufts of partial glomeruli
**Mean glomerular volume (Weibel-Gomes formula)** (S1,S2)	$Aglom^{3/2}\ x\ \frac{\beta}{d}$	*β=1.382 (shape coefficient for spheres)*<br>*d*= 1.01 (coefficient of adjustment on the size of the glomeruli)
**Volumetric Glomerular density** (S1)	$Dglom = \frac{1}{\beta}\ x\ \sqrt[2]{\frac{\left(\frac{number\ of\ non-sclerotic\ glomeruli}{cortical\ area}\right)^3}{\frac{total\ area\ of\ non-sclerotic\ glomeruli}{cortical\ area}}}$	*β=1.382 (shape coefficient for spheres)*<br>number of non-sclerotic glomeruli:<br>=1 if complete glomeruli<br>=0.5 if partial glomeruli
**Volumetric Intra-glomerular Cell density** (17)	$\frac{1}{\beta}\ x\ \sqrt[2]{\frac{\left(\frac{number\ of\ cells}{Total\ glomerular\ area}\right)^3}{\frac{total\ area\ of\ of\ cells}{Total\ glomerular\ area}}}$	*β=1.382 (shape coefficient for spheres)*
**Relative Area**	$100x\ \frac{Total\ Area\ of\ Object}{Cortical\ (or\ glomerular) Area}$	
**Tubular atrophy** (S3)	$\frac{number\ of\ atrophic\ tubule}{total\ tubule\ number}$	
**Interstitial area** (12)	*Cortical area-arteries area - veins area-tubules area - non sclerotic glomeruli area - globally sclerotic glomeruli area*	
**Interstitial fibrosis (%)** (S3)	$\frac{Interstitial\ area}{cortical\ area}$	
**Leukocyte volumetric density** (15)	$glom = \frac{1}{\beta}\ x\ \sqrt[2]{\frac{\left(\frac{number\ of\ Interstitial\ Leukocytes}{Interistial\ Area - Total\ capillary\ area}\right)^3}{\frac{total\ area\ of\ Interstitial\ Leukocytes}{nteristial\ Area - Total\ capillary\ area}}}$	*β=1.382 (shape coefficient for spheres)*
**Capillary Occlusion by leukocytes (%)**	$100x\ \frac{Total\ Area\ of\ Leukocytes\ in\ capillaries}{Total\ Capillaries\ Area}$	
**Intimal thickening (luminal stenosis)** (S4)	$1 - \frac{Aext - Aint}{Aext}$	Aint= area of internal elastic membrane<br>Aext= area of external elastic membrane

Supplemental Table S2: Prediction performance of the 5-year eGFR by Machine Learning models tested on 128 patients from the Training/Test cohort

Models	Morphometric, clinical and Biological Data<br>MAE ± SD	Morphometric Data and eGFR at Biopsy<br>MAE ± SD	Morphometric Data<br>MAE ± SD	Clinical and Biological Data<br>MAE ± SD
Bayesian	14.4±1.1	14.4±1.2	17.8±1.7	17.2±1.6
Elastic net	14.0±1.1	14.0±1.2	17.9±1.5	17.2±1.8
Gradient Boosting	15.5±2.1	15.3±2.1	18.9±2.3	17.4±2.1
Kernel Ridge	14.0±1.2	14.1±1.3	18.0±1.3	21.5±2.5
LASSO	14.0±1.1	14.0±1.2	17.9±1.6	17.3±1.9
OMP	13.6±1.3	13.6±1.3	18.5±2.0	17.3±1.9
Polynomial	25.2±5.6	15.5±1.6	20.0±2.9	17.3±2.1
Random Forest	14.7±2.0	14.7±2.0	18.6±2.1	17.5±2.2
Ridge	14.6±1.3	14.8±1.3	19.2±2.3	17.3±2.0
SVR	16.3±1.7	16.1±1.7	18.0±2.0	18.1±2.1
Multiple Linear	15.8± 1.3	15.5± 1.6	20.0±2.9	17.3±2.1

MAE: Mean Absolute Error in mL/min/1.73m2

SD: Standard Deviation

LASSO: Least Absolute Shrinkage and Selection Operator

OMP: Orthogonal Matching Pursuit Polynomial

SVR: Support Vector Regression

Supplemental Table S3: Prediction performance of the 7-year eGFR by Machine Learning models tested on 83 patients from the Training/Test cohort

Models	Morphometric, clinical and Biological Data<br>MAE ± SD	Morphometric Data and eGFR at Biopsy<br>MAE ± SD	Morphometric Data<br>MAE ± SD	Clinical and Biological Data<br>MAE ± SD
Bayesian	17.7±2.0	17.8±2.0	19.6±1.9	19.6 ± 1.5
Elastic net	15.2±3.5	14.8±2.6	18.3±2.4	19.5 ± 1.4
Gradient Boosting	16.3±2.2	16.4±2.2	20.0±2.8	21.0 ± 2.4
Kernel Ridge	15.1±3.2	14.9±2.9	19.8±3.4	25.2 ± 2.6
LASSO	15.2±3.6	14.7±2.8	18.3±2.4	19.5 ± 1.4
OMP	15.2±2.6	14.6±2.7	20.3±2.5	20.3 ± 2.1
Polynomial	20.3±4.9	17.1±2.1	19.9±1.6	20.7 ± 2.5
Random Forest	15.6±2.8	15.9±2.8	20.0±2.2	20.5 ± 2.4
Ridge	16.8±2.9	16.0±2.3	19.9±1.7	19.8 ± 1.7
SVR	18.5±2.6	18.2±2.8	20.2±2.5	20.7 ± 2.5
Multiple Linear	17.5±2.7	17.1±2.0	19.9±1.6	20.7 ± 2.5

MAE: Mean Absolute Error in mL/min/1.73m2

SD: Standard Deviation

LASSO: Least Absolute Shrinkage and Selection Operator

OMP: Orthogonal Matching Pursuit Polynomial

SVR: Support Vector Regression

Supplemental Table S4: Prediction performance of the 5-year eGFR by Machine Learning models tested on 202 patients from the Application cohort

	Morphometric, clinical and Biological Data			Morphometric Data and eGFR at Biopsy			Morphometric Data			Clinical and Biological Data		
**Models**	**MAE ± SD**	**r**	**p values**	**MAE ± SD**	**r**	**p values**	**MAE ± SD**	**r**	**p values**	**MAE ± SD**	**r**	**p values**
Bayesian	14.8±12.0	0.59	***<0.001***	15.1±12.1	0.58	***<0.001***	20.0±16.6	0.08	0.238	17.0±13.8	0.33	***<0.001***
Elastic net	15.3±12.0	0.59	***<0.001***	15.4±12.1	0.60	***<0.001***	21.4±17.1	0.20	***0.005***	17.2±13.8	0.33	***<0.001***
Gradient Boosting	14.9±12.0	0.52	***<0.001***	14.8±11.9	0.53	***<0.001***	16.9±14.2	0.21	***0.003***	16.9±14.0	0.30	***<0.001***
Kernel Ridge	20.2±14.1	0.57	***<0.001***	19.5±13.7	0.58	***<0.001***	25.3±19.6	0.15	***0.031***	20.6±17.1	0.16	***0.027***
LASSO	15.6±12.1	0.59	***<0.001***	15.6±12.1	0.59	***<0.001***	20.5±16.4	0.19	***0.006***	17.0±13.8	0.33	***<0.001***
OMP	17.2±12.9	0.60	***<0.001***	17.2±12.9	0.60	***<0.001***	20.7±16.6	0.23	***0.001***	17.7±13.9	0.30	***<0.001***
Polynomial	37.6±29.1	0.35	***<0.001***	15.8±12.8	0.56	***<0.001***	35.9±17.2	0.26	***<0.001***	17.4±14.0	0.32	***<0.001***
Random Forest	15.1±11.6	0.55	***<0.001***	15.2±11.6	0.55	***<0.001***	17.0±13.5	0.29	***<0.001***	18.3±14.9	0.29	***<0.001***
Ridge	19.0±13.5	0.58	***<0.001***	17.8±13.1	0.59	***<0.001***	23.4±18.2	0.19	***0.008***	17.3±13.9	0.32	***<0.001***
SVR	18.2±13.8	0.49	***<0.001***	16.0±13.3	0.48	***<0.001***	16.9±14.2	0.28	***<0.001***	16.7±13.7	0.29	**0.007**
Multiple Linear	17.4±13.5	0.55	***<0.001***	15.8±12.8	0.58	***<0.001***	23.4±17.5	0.26	***<0.001***	17.4±14.0	0.32	***<0.001***

MAE: Mean Absolute Error in mL/min/1.73m2

SD: Standard Deviation

LASSO: Least Absolute Shrinkage and Selection Operator

OMP: Orthogonal Matching Pursuit Polynomial

SVR: Support Vector Regression

Supplemental Table S5: Prediction performance of the 7-year eGFR by Machine Learning models tested on 189 patients from the Application cohort

	Morphometric, clinical and Biological Data			Morphometric Data and eGFR at Biopsy			Morphometric Data			Clinical and Biological Data		
**Models**	**MAE ± SD**	**r**	**p values**	**MAE ± SD**	**r**	**p values**	**MAE ± SD**	**r**	**p values**	**MAE ± SD**	**r**	**p values**
Bayesian	19.2±14.9	0.52	***<0.001***	19.3±14.9	0.52	***<0.001***	27.3±21.5	0.11	0.135	19.8±15.4	0.26	***<0.001***
Elastic net	18.6±14.2	0.54	***<0.001***	18.4±14.1	0.54	***<0.001***	28.1±22.6	0.18	***0.014***	19.9±15.4	0.26	***<0.001***
Gradient Boosting	16.6±14.5	0.49	***<0.001***	16.7±14.5	0.49	***<0.001***	18.9±16.0	0.21	***0.004***	19.2±15.2	0.25	***<0.001***
Kernel Ridge	19.2±14.8	0.51	***<0.001***	18.8±14.1	0.54	***<0.001***	27.9±22.2	0.17	***0.019***	25.8±20.2	0.1	0.293
LASSO	18.4±14.3	0.52	***<0.001***	18.5±14.0	0.55	***<0.001***	28.0±22.5	0.18	***0.013***	19.9±15.4	0.26	***<0.001***
OMP	17.7±14.0	0.54	***<0.001***	17.7±14.0	0.54	***<0.001***	28.9±23.0	0.18	***0.012***	20.1±15.4	0.25	***<0.001***
Polynomial	21.7±16.8	0.38	***<0.001***	20.8±15.7	0.47	***<0.001***	43.2±28.0	0.17	***0.019***	21.8±16.6	0.17	***0.020***
Random Forest	15.9±12.8	0.58	***<0.001***	16.0±12.8	0.58	***<0.001***	19.6±15.9	0.25	***<0.001***	20.1±15.7	0.22	***0.002***
Ridge	18.9±14.6	0.51	***<0.001***	18.1±13.8	0.54	***<0.001***	27.5±22.0	0.17	***0.016***	20.2±15.5	0.23	***0.001***
SVR	19.2±15.2	0.42	***<0.001***	21.1±16.1	0.47	***<0.001***	74.7±166.2	-0.1	0.204	19.3±15.7	0.18	***0.015***
Multiple Linear	26.0±18.9	0.40	***<0.001***	20.8±15.7	0.47	***<0.001***	43.2±28.0	0.17	***0.019***	21.8±16.6	0.17	***0.020***

MAE: Mean Absolute Error in mL/min/1.73m2

SD: Standard Deviation

LASSO: Least Absolute Shrinkage and Selection Operator

OMP: Orthogonal Matching Pursuit Polynomial

SVR: Support Vector Regression

Supplemental Table S6: Application cohort

	**Application cohort (n = 221)**	**Patients from Lille (n = 132)**	**Patients from Lyon (n = 89)**	**p value$^{\alpha}$**
**Clinical Data**				
Age (years)	52±14	52±14	51±13	0.418
Male sex, n (%)	142 (64)	87 (66)	55 (62)	0.650
History of Hypertension, n (%)	159 (72)	91 (69)	68 (76)	0.285
History of Diabetes, n (%)	38 (17)	27 (20)	11 (12)	0.146
**Biological Data**				
DSA at Biopsy, n (%)	26 (11)	26 (20)	0 (0)	**<0.001**
BK virus Replication at Biopsy, n (%)	10 (5)	3 (2)	7 (8)	0.094
eGFR at Biopsy (mL/min/1,73m^2)	53±18	53±19	53±19	0.809
3 years eGFR (mL/min/1,73m^2)	53±22	51±24	55±19	0.125
**Follow-up**				
Rejection at 3 years, n (%)	31 (14)	9 (7)	22 (25)	**<0.001**
Dialysis at 3 years, n (%)	5 (2)	4 (3)	1 (1)	0.650
**Morphometric Parameters$^{\beta}$**				
Non sclerotic Glomeruli (%)	94±9	92±9	94±9	**0.049**
Mean Glomerular Volume (µm^3)	6.3±2.7 x10^6	6.8±3.0 x10^6	5.5±2.0 x106	**0.002**
Glomerular Density (/mm$^{3\,*}$)	12±6	13±7	11±4	0.055
Artery luminal stenosis (%)	31±21	31±22	31±19	0.893
Mean Tubular Area (µm^2)	3131±887	2980±965	3355±704	**<0.001**
Tubular Atrophy (%)	21±9	25±9	16±6	**<0.001**
Interstitial Fibrosis (%)	22±6	22±6	21±5	0.100
Relative Peritubular Capillaries area (% of cortical Area)	8±1	8±2	7±1	**<0.001**
Mean Number of Leukocytes per Peritubular Capillary	0.1±0.1	0.1±0.1	0.1±0.1	0.999
Number of Leukocytes in the Most Affected Capillary	3±1	3±1	3±1	0.999
Capillary Occlusion by leukocytes (%)	2±1	2±1	2±1	0.999
Interstitial Leukocytes density (cells/mm^3 ¥)	224165±59606	216079±52663	236156±67157	**0.014**
Mean Mesangial Area (µm^2)	8399±2316	8188±2251	8712±2389	0.073
Mean Parietal cells area (µm^2)	42±5	43±5	42±4	0.196
Mean Parietal cells density (cells/mm$^{3\,\delta}$)	51127±21881	41521±16836	65374±20767	**<0.001**
Mean Podocytes area (µm^2)	43±5	42±5	44±5	**<0.001**
Mean Podocytes density (cells/mm$^{3\,\delta}$)	79267±26909	74163±22107	86838±31395	**0.002**
Mean Endothelial cells area (µm^2)	22±2	23±2	22±2	0.760
Mean Endothelial cells density (cells/mm^3 $^{\delta}$)	177167±60605	164202±65709	196395±46140	**<0.001**
Mean Mesangial cells area (µm^2)	24±3	24±3	25±2	**<0.001**
Mean Mesangial cells density (cells/mm^3 $^{\delta}$)	107919±42628	88008±32800	137451±38272	**<0.001**
Mean Glomerular Capillary Area (µm^2)	123±24	128±27	115±16	**<0.001**
Relative Capillary Area (% of Glomerulus)	26±5	27±5	25±5	**0.039**

DSA: Donor-Specific Antibodies

eGFR: estimated Glomerular Filtration Rate

Variables are expressed as mean±SD

α: Student's or Mann–Whitney's T-tests compared two quantitative unpaired variables (Lyon and Lille) depending on whether the distribution was normal or not. Categorical variables were compared using the χ² test (or Fisher's exact test when appropriate)

SUPPLEMENTAL FIGURE

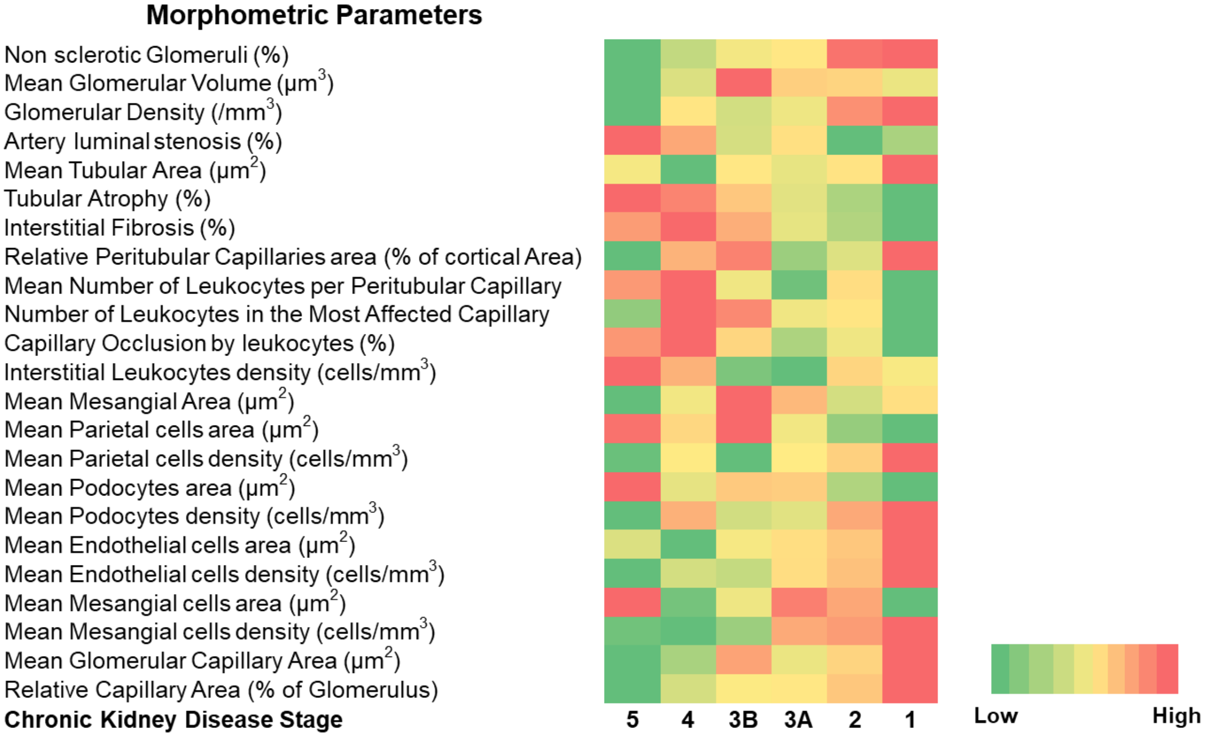

Supplemental Figure S1: Heatmap of histomorphometric parameters from the Training/Test cohort (n=146) according to the chronic kidney disease stage at three years

SUPPLEMENTAL REFERENCES

S1. Issa N, Lopez CL, Denic A, Taler SJ, Larson JJ, Kremers WK, Ricaurte L, Merzkani MA, Alexander MP, Chakkera HA, Stegall MD, Augustine JJ, Rule AD: Kidney Structural Features from Living Donors Predict Graft Failure in the Recipient. *J Am Soc Nephrol* 31: 415–423, 2020

S2. Weibel ER, Gomez DM: A principle for counting tissue structures on random sections. *Journal of Applied Physiology* 17: 343–348, 1962

S3. Hermsen M, Bel T de, Boer M den, Steenbergen EJ, Kers J, Florquin S, Roelofs JJTH, Stegall MD, Alexander MP, Smith BH, Smeets B, Hilbrands LB, Laak JAWM van der: Deep Learning–Based Histopathologic Assessment of Kidney Tissue. *JASN* 30: 1968–1979, 2019

S4. Merzkani MA, Denic A, Narasimhan R, Lopez CL, Larson JJ, Kremers WK, Chakkera HA, Park WD, Taler SJ, Stegall MD, Alexander MP, Issa N, Rule AD: Kidney Microstructural Features at the Time of Donation Predict Long-term Risk of Chronic Kidney Disease in Living Kidney Donors. *Mayo Clin Proc* 96: 40–51, 2021

Section & Topic	No	Item	Reported on page #
**TITLE OR ABSTRACT**			
	**1**	Identification as a study of diagnostic accuracy using at least one measure of accuracy (such as sensitivity, specificity, predictive values, or AUC)	1
**ABSTRACT**			
	**2**	Structured summary of study design, methods, results, and conclusions (for specific guidance, see STARD for Abstracts)	2
**INTRODUCTION**			
	**3**	Scientific and clinical background, including the intended use and clinical role of the index test	3-4
	**4**	Study objectives and hypotheses	4
**METHODS**			
Study design	**5**	Whether data collection was planned before the index test and reference standard were performed (prospective study) or after (retrospective study)	5
Participants	**6**	Eligibility criteria	5
	**7**	On what basis potentially eligible participants were identified (such as symptoms, results from previous tests, inclusion in registry)	5
	**8**	Where and when potentially eligible participants were identified (setting, location and dates)	5
	**9**	Whether participants formed a consecutive, random or convenience series	5
Test methods	**10a**	Index test, in sufficient detail to allow replication	6-10
	**10b**	Reference standard, in sufficient detail to allow replication	6-10
	**11**	Rationale for choosing the reference standard (if alternatives exist)	9-10
	**12a**	Definition of and rationale for test positivity cut-offs or result categories of the index test, distinguishing pre-specified from exploratory	9-10
	**12b**	Definition of and rationale for test positivity cut-offs or result categories of the reference standard, distinguishing pre-specified from exploratory	9-10
	**13a**	Whether clinical information and reference standard results were available to the performers/readers of the index test	9-10
	**13b**	Whether clinical information and index test results were available to the assessors of the reference standard	9-10
Analysis	**14**	Methods for estimating or comparing measures of diagnostic accuracy	7-10
	**15**	How indeterminate index test or reference standard results were handled	NA
	**16**	How missing data on the index test and reference standard were handled	9-10
	**17**	Any analyses of variability in diagnostic accuracy, distinguishing pre-specified from exploratory	NA
	**18**	Intended sample size and how it was determined	7
**RESULTS**			
Participants	**19**	Flow of participants, using a diagram	Figure 1
	**20**	Baseline demographic and clinical characteristics of participants	11-12 and Table 1
	**21a**	Distribution of severity of disease in those with the target condition	11-12 and Table 2
	**21b**	Distribution of alternative diagnoses in those without the target condition	11-12 and Table 2
	**22**	Time interval and any clinical interventions between index test and reference standard	NA
Test results	**23**	Cross tabulation of the index test results (or their distribution) by the results of the reference standard	12-15 Table 3 and 4
	**24**	Estimates of diagnostic accuracy and their precision (such as 95% confidence intervals)	12-15
	**25**	Any adverse events from performing the index test or the reference standard	NA
**DISCUSSION**			
	**26**	Study limitations, including sources of potential bias, statistical uncertainty, and generalisability	16-21
	**27**	Implications for practice, including the intended use and clinical role of the index test	16-21
**OTHER INFORMATION**			
	**28**	Registration number and name of registry	NA
	**29**	Where the full study protocol can be accessed	NA
	**30**	Sources of funding and other support; role of funders	None

cc BY SA

STARD 2015

AIM

STARD stands for "Standards for Reporting Diagnostic accuracy studies". This list of items was developed to contribute to the completeness and transparency of reporting of diagnostic accuracy studies. Authors can use the list to write informative study reports. Editors and peer-reviewers can use it to evaluate whether the information has been included in manuscripts submitted for publication.

EXPLANATION

A **diagnostic accuracy study** evaluates the ability of one or more medical tests to correctly classify study participants as having a **target condition.** This can be a disease, a disease stage, response or benefit from therapy, or an event or condition in the future. A medical test can be an imaging procedure, a laboratory test, elements from history and physical examination, a combination of these, or any other method for collecting information about the current health status of a patient.

The test whose accuracy is evaluated is called **index test.** A study can evaluate the accuracy of one or more index tests. Evaluating the ability of a medical test to correctly classify patients is typically done by comparing the distribution of the index test results with those of the **reference standard**. The reference standard is the best available method for establishing the presence or absence of the target condition. An accuracy study can rely on one or more reference standards.

If test results are categorized as either positive or negative, the cross tabulation of the index test results against those of the reference standard can be used to estimate the **sensitivity** of the index test (the proportion of participants *with* the target condition who have a positive index test), and its **specificity** (the proportion *without* the target condition who have a negative index test). From this cross tabulation (sometimes referred to as the contingency or "2x2" table), several other accuracy statistics can be estimated, such as the positive and negative **predictive values** of the test. Confidence intervals around estimates of accuracy can then be calculated to quantify the statistical **precision** of the measurements.

If the index test results can take more than two values, categorization of test results as positive or negative requires a **test positivity cut-off**. When multiple such cut-offs can be defined, authors can report a receiver operating characteristic (ROC) curve which graphically represents the combination of sensitivity and specificity for each possible test positivity cut-off. The **area under the ROC curve** informs in a single numerical value about the overall diagnostic accuracy of the index test.

The **intended use** of a medical test can be diagnosis, screening, staging, monitoring, surveillance, prediction or prognosis. The **clinical role** of a test explains its position relative to existing tests in the clinical pathway. A replacement test, for example, replaces an existing test. A triage test is used before an existing test; an add-on test is used after an existing test.

Besides diagnostic accuracy, several other outcomes and statistics may be relevant in the evaluation of medical tests. Medical tests can also be used to classify patients for purposes other than diagnosis, such as staging or prognosis. The STARD list was not explicitly developed for these other outcomes, statistics, and study types, although most STARD items would still apply.

DEVELOPMENT

This STARD list was released in 2015. The 30 items were identified by an international expert group of methodologists, researchers, and editors. The guiding principle in the development of STARD was to select items that, when reported, would help readers to judge the potential for bias in the study, to appraise the applicability of the study findings and the validity of conclusions and recommendations. The list represents an update of the first version, which was published in 2003.

More information can be found on [http://www.equator-network.org/reporting-guidelines/stard.](http://www.equator-network.org/reporting-guidelines/stard)

BY SA
